# Patient Outcomes and Lessons Learned From Treating Patients With Severe COVID-19 at a Long-term Acute Care Hospital: Single-Center Retrospective Study

**DOI:** 10.2196/31502

**Published:** 2022-02-10

**Authors:** Pete Grevelding, Henry Charles Hrdlicka, Steve Holland, Lorraine Cullen, Amanda Meyer, Catherine Connors, Darielle Cooper, Allison Greco

**Affiliations:** 1 Milne Institute for Healthcare Innovation Gaylord Specialty Healthcare Wallingford, CT United States; 2 Clinical and Medical Services Gaylord Specialty Healthcare Wallingford, CT United States; 3 Department of Radiology Services Gaylord Specialty Healthcare Wallingford, CT United States; 4 Department of Inpatient Respiratory Therapy Gaylord Specialty Healthcare Wallingford, CT United States; 5 Department of Inpatient Occupational Therapy Gaylord Specialty Healthcare Wallingford, CT United States; 6 Department of Inpatient Physical Therapy Gaylord Specialty Healthcare Wallingford, CT United States; 7 Department of Inpatient Speech Language Pathology Gaylord Specialty Healthcare Wallingford, CT United States

**Keywords:** COVID-19, SARS-CoV-2, post–COVID-19, subacute COVID-19, postacute care, long-term acute care hospital, pulmonary, speech therapy, speech-language pathology, rehabilitation, physical therapy, occupational therapy, respiratory therapy

## Abstract

**Background:**

With the continuation of the COVID-19 pandemic, shifting active COVID-19 care from short-term acute care hospitals (STACHs) to long-term acute care hospitals (LTACHs) could decrease STACH census during critical stages of the pandemic and maximize limited resources.

**Objective:**

This study aimed to describe the characteristics, clinical management, and patient outcomes during and after the acute COVID-19 phase in an LTACH in the Northeastern United States.

**Methods:**

This was a single-center group comparative retrospective analysis of the electronic medical records of patients treated for COVID-19–related impairments from March 19, 2020, through August 14, 2020, and a reference population of medically complex patients discharged between December 1, 2019, and February 29, 2020. This study was conducted to evaluate patient outcomes in response to the holistic treatment approach of the facility.

**Results:**

Of the 127 total COVID-19 admissions, 118 patients were discharged by the data cutoff. At admission, 29.9% (38/127) of patients tested positive for SARS-CoV-2 infection. The mean age of the COVID-19 cohort was lower than that of the reference cohort (63.3, 95% CI 61.1-65.4 vs 65.5, 95% CI 63.2-67.8 years; *P*=.04). There were similar proportions of males and females between cohorts (*P*=.38); however, the proportion of non-White/non-Caucasian patients was higher in the COVID-19 cohort than in the reference cohort (odds ratio 2.79, 95% CI 1.5-5.2; *P*=.001). The mean length of stay in the COVID-19 cohort was similar to that in the reference cohort (25.5, 95% CI 23.2-27.9 vs 29.9, 95% CI 24.7-35.2 days; *P*=.84). Interestingly, a positive correlation between patient age and length of stay was observed in the COVID-19 cohort (r^2^=0.05; *P*=.02), but not in the reference cohort. Ambulation assistance scores improved in both the reference and COVID-19 cohorts from admission to discharge (*P*<.001). However, the mean assistance score was greater in the COVID-19 cohort than in the reference cohort at discharge (4.9, 95% CI 4.6-5.3 vs 4.1, 95% CI 3.7-4.7; *P*=.001). Similarly, the mean change in gait distance was greater in the COVID-19 cohort than in the reference cohort (221.1, 95% CI 163.2-279.2 vs 146.4, 95% CI 85.6-207.3 feet; *P*<.001). Of the 16 patients mechanically ventilated at admission, 94% (15/16) were weaned before discharge (mean 11.3 days). Of the 75 patients admitted with a restricted diet, 75% (56/75) were discharged on a regular diet.

**Conclusions:**

The majority of patients treated at the LTACH for severe COVID-19 and related complications benefited from coordinated care and rehabilitation. In comparison to the reference cohort, patients treated for COVID-19 were discharged with greater improvements in ambulation distance and assistance needs during a similar length of stay. These findings indicate that other patients with COVID-19 would benefit from care in an LTACH.

## Introduction

Patients hospitalized with severe COVID-19 caused by SARS-CoV-2 infection may face a long hospital length of stay (LOS), making it unreasonable to expect a direct discharge to home [1]. Indeed, COVID-19 is predicted to result in significant morbidity for some patients, with the need for medical and rehabilitation services for 6 months or longer after the initial diagnosis [2].

Long-term acute care hospitals (LTACHs) can provide these postacute care and rehabilitation services in the post-COVID phase. They can also provide an alternative to conventional short-term acute care hospitals (STACHs) for active COVID-19 treatment, thereby reducing the burden on the STACH system when resources are already limited [3,4].

LTACHs are certified acute care hospitals equipped to provide long-term (average LOS of 25-28 days) acute level care to medically complex patients. LTACHs are able to treat patients who require a higher level of care than what other rehabilitation facilities may be able to provide. Medically complex patients are often transferred to the LTACH setting as soon as they are found to be hemodynamically stable. Once at the LTACH, an interdisciplinary care plan, including continued treatment for underlying conditions and targeted holistic rehabilitation, is started. While it is the hope that each patient is able to be discharged to home, patients may also be transferred to other facilities such as skilled nursing facilities to continue their recovery if necessary.

It has been proposed that patients with severe COVID-19 may benefit from the inpatient respiratory, functional, and neurological rehabilitation provided at LTACHs [5]. Early rehabilitation may also reduce disability and improve clinical outcomes in patients with COVID-19 [6-9].

Here, we report on patient characteristics, clinical management strategies, and patient outcomes from an LTACH caring for patients with severe COVID-19, as well as make comparisons with the typical medical population cared for at the LTACH.

## Methods

### Study Design, Setting, and Study Population

This retrospective study was conducted at Gaylord Specialty Healthcare, a rehabilitation-focused LTACH in the Northeastern United States. COVID-19–related data were collected from March 19, 2020, through August 14, 2020. The study data are for 117 individuals who were treated in regional STACHs for acute COVID-19 and then discharged to the LTACH for post–COVID-19 care and rehabilitation. Due to STACH readmissions for acute decompensation, 8 of the 117 individuals accounted for 10 additional admissions, for a total of 127 admissions. Of the 127 total admissions/readmissions, there were 118 total discharges by the data cutoff of August 14, 2020, with 9 admissions remaining as active patients.

Data from a historical reference cohort (control population) consisting of 157 individuals discharged from December 1, 2019, through February 29, 2020, were also collected. Similar to the COVID-19 cohort, some individuals required temporary readmission to a STACH before being readmitted to the LTACH setting. Ten of the 157 individuals accounted for 13 additional readmissions in the reference cohort, for a total of 170 admissions and discharges. Although this led to uneven population sizes, the 2-month time frame for the historical control was selected to normalize potential seasonal variances and minimize the effect of annual regulatory and insurance changes.

When describing patient demographics, we compared the 117 individual patients admitted for COVID-19–related rehabilitation and the 157 individuals admitted during the reference time frame. When comparing LOS, we used the LOS for the 118 total COVID-19 discharges and the LOS for the 170 total reference cohort discharges. For all other comparisons, we used data from the total admissions or subpopulations.

### Protocols for Patients With Confirmed or Suspected SARS-CoV-2 Infection

Similar to arrangements made by other LTACH facilities with regional hospitals, patients who required postacute care for COVID-19–related issues and those who were still SARS-CoV-2 positive were accepted from STACHs to help unburden those facilities [10]. Additionally, when available beds in the LTACH facility were scarce, health care workers and other first responders were prioritized for admission to ensure other regional health care facilities were able to be adequately staffed during the pandemic.

Patients with active or prior SARS-CoV-2 infection were housed on separate floors of the hospital, similar to the practical arrangements of other postacute care facilities [11]. Patients with confirmed or suspected SARS-CoV-2 infection were housed in negative-pressure rooms or in rooms with portable or ceiling-mounted air scrubbers.

Personal protective equipment protocols for the COVID-19 cohort included the use of face shields, N95 particulate respirator masks or duck bill surgical masks, scrub caps, and boot covers, as well as uniform laundering at an outside facility. Powered air-purifying respirators were available if needed. Due to a facility shortage of N95 respirator masks (ie, unknown/unstable resupply chains), these masks were sterilized for reuse by an outside facility.

To decrease personnel exposure to patients with suspected or confirmed SARS-CoV-2 infection and conserve personal protective equipment, we developed multidisciplinary “COVID-19 teams” responsible for patient isolation, testing, implementation of droplet precautions, and cluster care. Further, a dedicated respiratory therapist and intubation box were used to treat patients with active SARS-CoV-2 infection requiring mechanical ventilation or having a tracheostomy.

### Typical Care for Patients With a Pulmonary Condition

Using standardized measures and functional assessments, interdisciplinary clinical teams evaluated patients to determine functional impairments at admission. When applicable, a speech-language pathologist assessed patients for voicing, swallowing, and cognitive-communication impairments. Patients were mobilized throughout the day, including chair positioning of the bed, transfer to a bedside chair, and other exercises/ambulation as appropriate.

Within 24 hours of admission, patients with a tracheostomy were assessed for in-line speaking valve use. As patients progressed with the speaking valve, they were transitioned to tracheostomy capping and placed on the decannulation protocol (Multimedia Appendix 1). When appropriate, patients being mechanically ventilated were considered for the ventilator weaning protocol (Multimedia Appendix 2). Interdisciplinary rounds occurred weekly for patients being mechanically ventilated.

#### COVID-19–Specific Respiratory Therapy Considerations

SARS-CoV-2–positive patients completed self-directed exercises in their rooms, were seen for individual or co-treatment sessions in their rooms, and, once SARS-CoV-2 negative, participated in group pulmonary exercise therapy and education classes.

Patients who were desaturating or acutely decompensating were placed in the prone position by a multidisciplinary team (including physical therapy, nursing, and respiratory therapy). Prior to placing patients in the prone position, staff participated in training sessions on how to safely prone and reposition patients, manage leads and lines, and perform cardiopulmonary resuscitation while in the prone position. Patients who were functionally capable or were previously placed in the prone position during acute care, were educated on how to safely put themselves in the prone position and encouraged to do so when appropriate.

### Speech-Language Pathology

Many patients in the COVID-19 cohort presented with cognitive-communication deficits, potentially as a result of COVID-19–induced hypoxia, prolonged intubation, or sedation [12]. When appropriate, cognitive-communication assessments were performed by a speech-language pathologist on the COVID-19 team. Using tools, such as the Bioness Integrated Therapy System, worksheets, and group therapy sessions, speech-language pathology sessions focused on attention, memory, functional skills, and compensatory strategy use. The National Outcomes Measurement System (NOMS) assessment was used to summarize the overall cognitive communication status of the COVID-19 cohort at admission and discharge. The NOMS scale was developed by the American Speech-Language-Hearing Association, and consists of 15 functional communication measures used for adult health care [13-15]. These 15 measures were designed to describe functional abilities over time and to be diagnosis specific, meaning that patients would only be given the measures specific to their case. These measures are scored using a 7-level system based on speech-language pathologist clinical observations of the individual’s communication and swallowing ability. The diagnosis-specific functional communication measures used to describe the patients treated for postacute COVID-19 in this study included the following: attention, memory, problem solving, spoken language comprehension, spoken language expression, swallowing, and voice following tracheostomy.

For the purpose of this study, the functional communication measure scores were used to assign an overall cognitive-communication status, including the following: unable to assess, profound impairment, severe impairment, moderate-severe impairment, moderate impairment, mild-moderate impairment, mild impairment, within functional limits, or baseline. To facilitate statistical analysis, these statuses were then given a numerical value ranging from 1 (unable to assess) to 9 (baseline cognition). Due to the retrospective nature of the study, similar values were not readily available for the reference cohort and were not included.

Due to the correlation between prolonged intubation and dysphagia, speech-language pathology interventions also targeted swallowing dysfunction [16]. Dysphagia management comprises several aerosol-generating procedures, including oral mechanism examination, cough testing, reflexive cough, swallowing trials, and secretion management. Given the proximity and prolonged exposure to aerosols during instrumental evaluations and the need for multiple staff members, procedures, such as fiberoptic endoscopic evaluation of swallow and modified barium swallow study, were minimized. Thus, speech-language pathologists heavily relied on clinical swallowing evaluations for patients with active SARS-CoV-2 infection. Additionally, some patients in the COVID-19 cohort consented to performing clinical swallowing evaluations via telehealth to reduce potential SARS-CoV-2 exposure and transmission.

### Gait/Functional Status Assessment and Rehabilitation

At admission, physical therapists evaluated patient ambulatory status by assessing functional ability and gait distance. Patients received standard individualized physical therapy, and their gait quality and distance were challenged for progression as tolerated. Hypotension or tachycardia was present in some patients in the COVID-19 cohort. For these individuals, therapy was aimed at improving tolerance and progression. Functional Independence Measure (FIM) assistance level scores and gait distance were used to describe the functional ability of patients throughout recovery. With a mean interrater reliability ranging from 0.89 to 1.00, the FIM is a 2-domain (ie, motor function and cognitive function) 18-item assessment. This measure uses a 7-point ordinal scale to measures the amount of assistance provided by the therapist during treatment [17-20]. Here, we are only reporting the assistance level associated with the FIM scores for ambulation under the locomotion subscale [17-20]. The assistance level associated with FIM was analyzed separate to the gait distance component. We felt that analyzing assistance and gait distance separately was the best way to look at the functional status of the subject amidst infection control restrictions, potentially limiting patients to their rooms and potentially limiting ambulation distances.

### Statistical Analysis

Data were analyzed using GraphPad Prism version 9.0.0 (GraphPad Software). Prior to analysis, data were tested for normality using the Shapiro-Wilk test. Each data set was found to have one or more nonnormally distributed groups, and nonparametric tests were used accordingly. For hypothesis testing between 2 unpaired groups, the Mann-Whitney rank comparison test was conducted. For paired 2-group testing, the Wilcoxon matched-pairs signed-rank test was conducted. For hypothesis testing between 3 groups, the Kruskal-Wallis analysis of variance (ANOVA) test with the Dunn multiple comparison posthoc test was conducted.

To compare the proportions of racial demographics of the reference and COVID-19 cohorts, we subdivided the individuals into either self-reported White/Caucasian or non-White/non-Caucasian (Black/African American, Asian, bi/multiracial) racial demographics. The rationale behind this was 2-fold. First, since the start of the pandemic, there has been a reported disparity in the number of White and non-White individuals being infected with SARS-CoV-2. By comparing these proportions, we wished to determine if this was also reflected in our population. Second, due to their lower representation in our population, individuals who self-reported as bi/multiracial in the COVID-19 cohort did not meet the criteria to conduct reliable chi-square testing across more than 2 groups. We were left with the option to either exclude these individuals or combine them. While combining Asian and bi/multiracial individuals into 1 category would have worked, we opted to combine and compare all non-White/non-Caucasian (Black/African American, Asian, bi/multiracial) individuals to White/Caucasian individuals. Doing this also allowed us to conduct the Fisher exact test, which is preferred to the approximation calculated with chi-square testing. Additionally, the odds ratios (ORs) for the proportions and the respective 95% CIs (Baptiste Pike testing) were calculated. Moreover, the Fisher exact test, ORs, and Baptiste Pike test were used to compare the proportions of male and female individuals between the cohorts.

Nonlinear regression analysis was conducted to determine the correlation between 2 conditions using least-squares regression; 95% CIs are reported. An extra sum-of-squares F test was performed to evaluate the calculated slope of each regression against a hypothetical slope of 0.

When data from multiple time points and two or more groups were present, a 2-way mixed effects model ANOVA was used. This was to evaluate for the presence or absence of time effects independent of the cohort, cohort effects independent of time, and the effects of time and cohort combined. The Šídák multiple comparisons test was then used to calculate all in-group and between-group comparisons. Included in this analysis were the admission and discharge values for ambulation assistance (ie, FIM scores) and gait distance travelled. Changes in FIM scores and gait distance were compared using Mann-Whitney *U* tests.

### Ethics Approval

This study was written in compliance with our institutional privacy policy, the Health Insurance Portability and Accountability Act, and the standards set by the Declaration of Helsinki. Prior to beginning, this retrospective study was reviewed and given an exempt status by the Gaylord Specialty Healthcare Institutional Review Board.

## Results

### Patient Demographics

**Figure 1:**
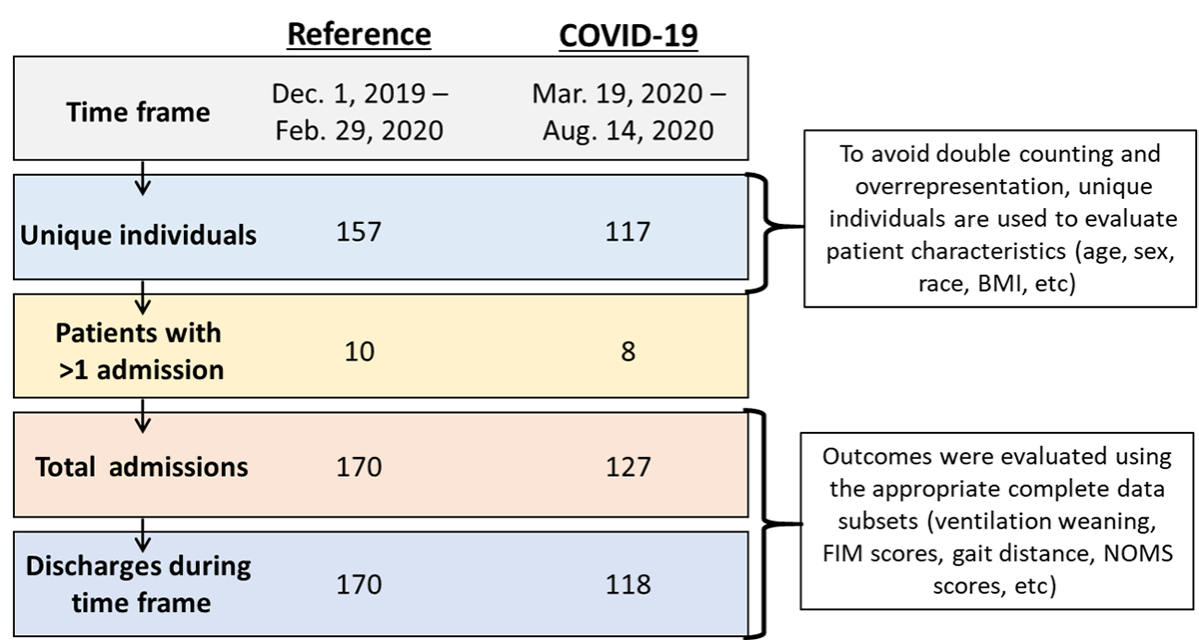
Study cohorts (COVID-19 cohort and reference cohort). FIM: Functional Independence Measure; NOMS: National Outcomes Measurement System.

**Figure 2:**
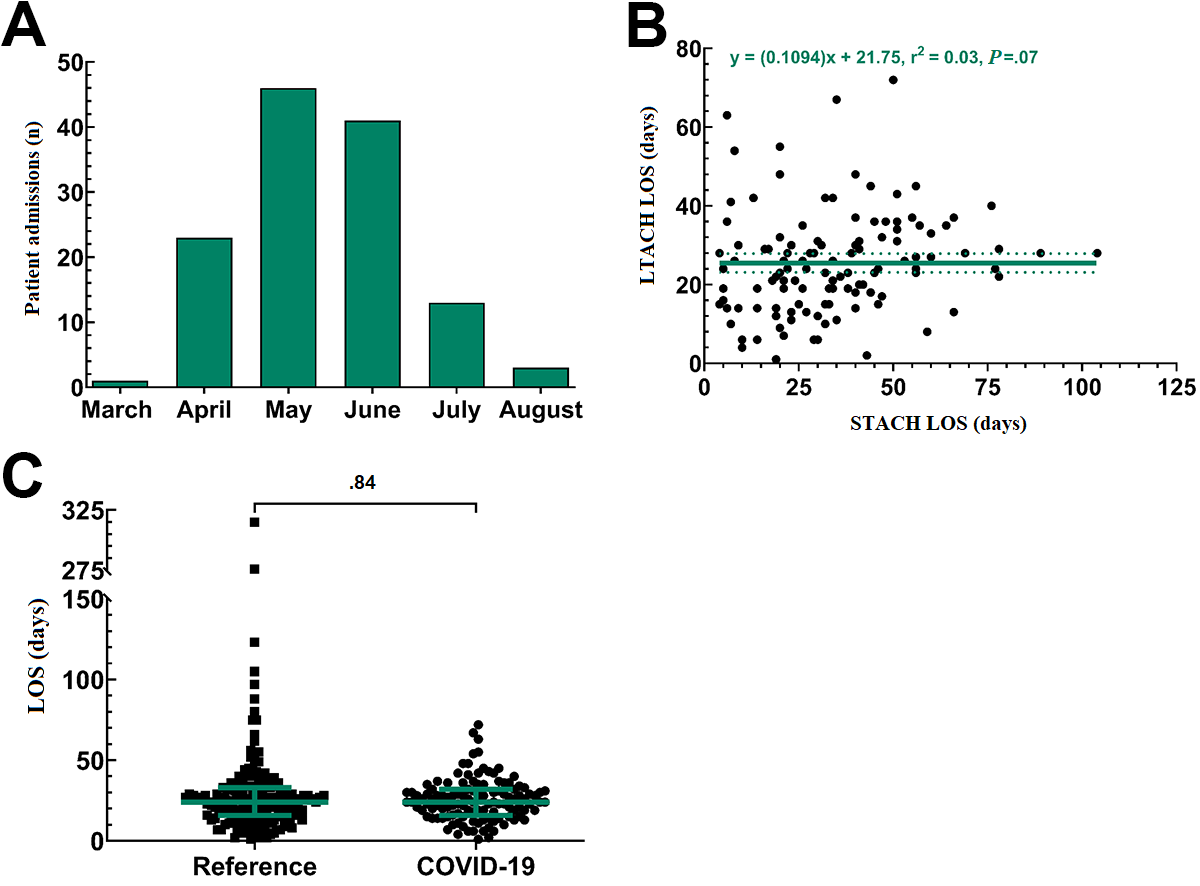
Trends in patient admission and length of stay (LOS) during the COVID-19 pandemic. (A) Patient admission from March 19, 2020, to August 14, 2020. (B) Nonlinear regression analysis for the correlation between patient long-term acute care hospital (LTACH) LOS and short-term acute care hospital (STACH) LOS. The solid regression line shows the correlation coefficient, and the dotted lines show the 95% CI. (C) Scatter plot for the comparison of the LTACH LOS between the reference and COVID-19 cohorts. The colored lines represent the median and interquartile range.

During the study period, 117 individuals, accounting for 127 total admissions, were admitted for COVID-19 or post–COVID-19 care as described above (Figure 1). COVID-19 admissions first peaked during May 2020 (Figure 2A), approximately 4 weeks later than in the New England/New York City area [21,22]. Of the 127 total admissions/readmissions, there were 118 total discharges by the data cutoff, with 9 admissions still receiving care. The COVID-19 cohort represented 17.2% (127/737) of the hospital census during the 4.5-month/148-day study period. For the 127 total COVID-19 admissions, the mean STACH LOS prior to LTACH admission or readmission was 34.3 (95% CI 30.6-37.9) days. The mean LTACH LOS for the 118 total discharges was 25.5 (95% CI 23.2-27.9) days. Regression analysis indicated that there was no correlation between STACH LOS and LTACH LOS (r^2^=0.03, *P*=.09; Figure 2B). Further, the mean COVID-19 cohort LOS was similar to the reference cohort LOS of 29.9 (95% CI 24.7-35.2) days (*P*=.84; Figure 2C).

**Figure 3:**
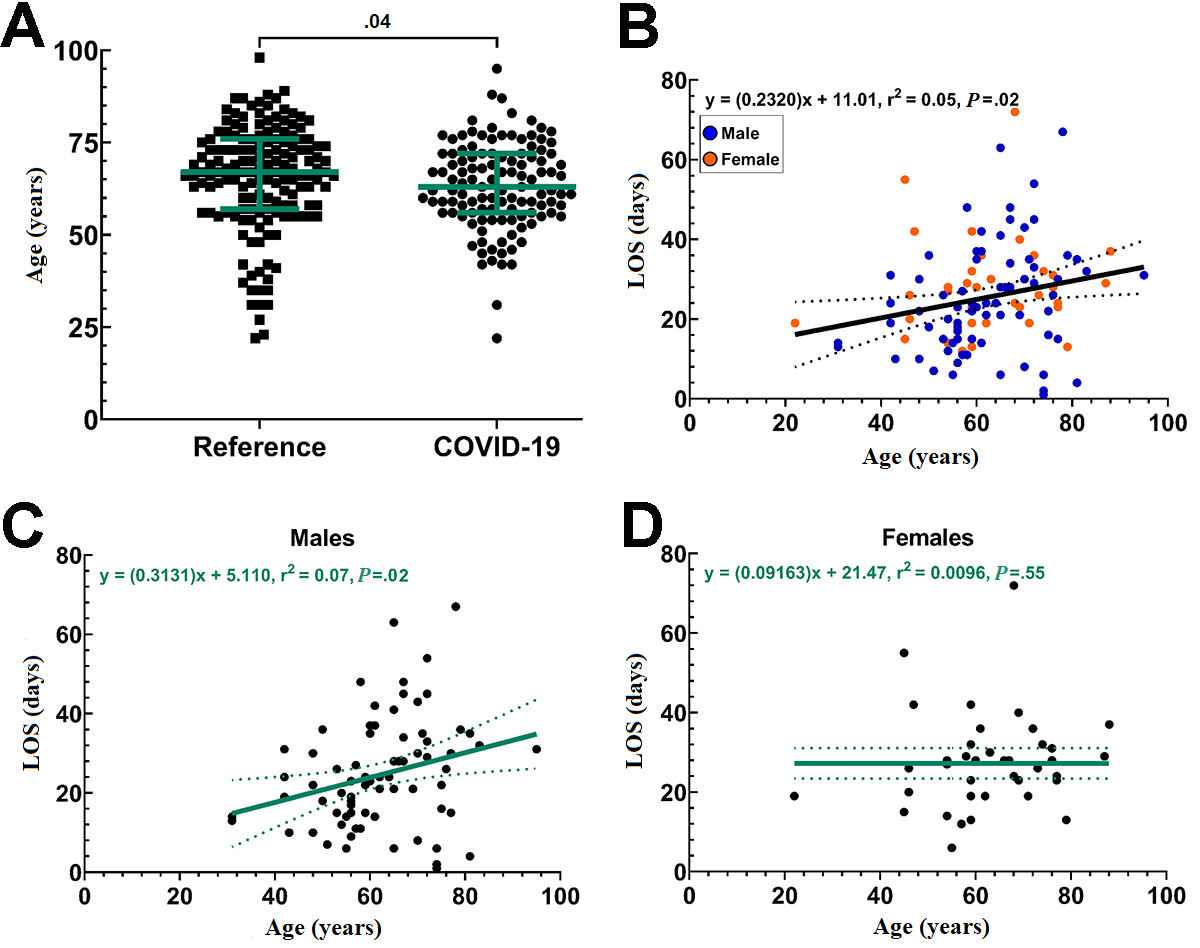
Age as a risk factor for prolonged COVID-19 illness. (A) Scatter plot showing the age distribution in the reference and COVID-19 cohorts. The colored lines represent the median and interquartile range. (B) Nonlinear regression analysis showing the correlation between patient age and long-term acute care hospital length of stay (LOS) in the overall COVID-19 cohort. Solid regression lines show the correlation coefficient surrounded by the 95% CI as dotted lines. (C, D) When evaluated by sex, this pattern was also observed in COVID-19 males alone (C), but was not present in COVID-19 females alone (D). Solid regression lines show the correlation coefficient surrounded by the 95% CI as dotted lines.

Compared to the reference cohort (n=157 individual patients), the COVID-19 cohort (n=117 individual patients) had a similar ratio of males to females (OR 0.79, 95% CI 0.49-1.3; *P*=.38), was younger (difference of medians=−4.0, 95% CI −6.0 to 0.0; *P*=.04; Figure 3A; Table 1), and had a greater representation of non-White racial demographics (32.5% vs 15.9%; OR 2.79, 95% CI 1.5-5.2; *P*=.001; Table 1). At admission, the most prevalent comorbidities in the COVID-19 cohort were hypertension (53.0%), hyperlipidemia (42.6%), dysphagia (38.3%), and type II diabetes mellitus (35.7%; Table 1).

At discharge, the most common discharge destinations of the reference cohort included home with health services (53/170, 31.2%), skilled nursing facility (43/170, 25.3%), emergent transfer to a STACH (38/170, 22.4%), and home without health services (11/170, 6.5%). The COVID-19 cohort discharge destinations were similar in nature and included home with health services (58/127, 45.7%), skilled nursing facility (35/127, 32.5%), home without health services (18/127, 14.2%), and emergent transfer to a STACH (14/127, 11.0%) (Table 1). Using chi-square testing, the distributions of the discharge destinations of the 2 cohorts were compared, and it was observed that the distributions were significantly different (χ^2^_5_=21.93; *P*<.001).

**Table 1:** Patient demographics and comorbidities at long-term acute care hospital admission.

Characteristic				Reference cohort^a^		COVID-19 cohort^b^		Group difference (95% CI) or chi square (*df*)		*P* value
Cohort age (years), mean (95% CI), n^c^				65.5 (63.2 to 67.8), 157		63.3 (61.1 to 65.4), 117		−4.0 (−6.0 to 0.0)^d^		.04
**Sex**								0.79 (0.49 to 1.3)^e^		.38
	Male, n^c^ (%)		92 (58.6)		75 (64.1)					
	Female, n^c^ (%)		65 (41.4)		42 (35.9)					
Male age (years), mean (95% CI), n^c^				64.0 (61.3 to 66.8), 92		63.2 (60.5 to 65.8), 75		−4.0 (−6.0 to 2.0)^d^		.30
Female age (years), mean (95% CI), n^c^				67.6 (63.7 to 71.6), 65		63.5 (59.5 to 67.4), 42		−6.5 (−11.0 to 0.0)^d^		.04
BMI (kg/m^2^), mean (95% CI), n^c^				27.2 (26.0 to 28.4), 157		29.9 (28.7 to 31.2), 117		3.2 (1.3 to 4.5)^d^		<.001
Length of stay (days), mean (95% CI), n^c^				29.9 (24.7 to 35.2), 170		25.5 (23.2 to 27.9), 118		0.0 (−3.0 to 3.0)^d^		.84
**Race^f^, n^c^(%)**								2.79 (1.5 to 5.2)^e^		.001
	White/Caucasian		132 (84.1)		79 (67.5)					
	**Non-White/non-Caucasian**		21 (15.9)		35 (32.5)					
		Black/African American	15 (9.2)		27 (23.7)					
		Asian	4 (2.4)		7 (6.1)					
		Bi/multiracial	2 (1.2)		1 (0.9)					
**Discharge destination^g^, n^c^(%)**								21.93 (*df* 5)		<.001^e,h^
	Home with health services		53 (31.2)		58 (45.7)					
	Skilled nursing facility		43 (25.3)		25 (19.7)					
	Home without health services		11 (6.5)		18 (14.2)					
	Emergent transfer to an ACH^i^		38 (22.4)		14 (11.0)					
	Planned transfer to an ACH		8.8 (8.8)		2 (1.6)					
	**Other**		10 (5.9)		10 (7.9)					
		Acute rehabilitation	0 (0.0)		1 (0.8)					
		Hospice/palliative care	6 (3.5)		0 (0.0)					
		Deceased	4 (2.4)		0 (0.0)					
		Patient at data cutoff	0 (0.0)		9 (7.1)					
**COVID-19 cohort comorbid conditions at LTACH^j^ admission^k^, n (%)**								N/A^l^		N/A
		Primary hypertension	N/A		61 (53.0)					
		Hyperlipidemia	N/A		49 (42.6)					
		Dysphagia	N/A		44 (38.3)					
		Type II diabetes mellitus	N/A		41 (35.7)					
		Acute kidney failure	N/A		25 (21.7)					
		Urinary tract infection	N/A		22 (19.1)					
		Severe obesity	N/A		14 (12.2)					

^a^The reference cohort included medically complex patients cared for at the facility from December 1, 2019, to February 29, 2020. Data from 170 admissions, consisting of 157 individuals, were included.

^b^The COVID-19 cohort included all COVID-19–related admissions from March 19, 2020, through August 14, 2020. Data from 127 admissions, consisting of 117 individuals, were included; 118 of the 127 admission cases were discharged by the data cutoff.

^c^The listed “n” value indicates the sample size analyzed to obtain each of the reported *P* values.

^d^Nonparametric Mann-Whitney test is used; group difference and reported 95% CI are based on differences of the medians.

^e^Fisher exact test is used to compare proportions of the self-reported demographics by group; group difference and reported 95% CI are calculated using odds ratios and Baptiste-Pike testing.

^f^Breakdown of self-reported demographics. For analysis of race-related demographics, groups were divided as either White/Caucasian or non-White/non-Caucasian. Individuals who elected to not report were not included in this analysis.

^g^Breakdown of recorded discharge destinations for all admissions in both the reference (n=170) and COVID-19 (n=127) cohorts. For analysis, the destinations of acute care hospital rehabilitation, hospice/palliative care, deceased, and patient at data cutoff were grouped.

^h^Chi-square testing was used to compare the distribution of discharge destinations for both cohorts.

^i^ACH: acute care hospital.

^j^LTACH: long-term acute care hospital.

^k^Comorbid conditions in the COVID-19 cohort were identified by International Classification of Diseases 10th revision (ICD-10) diagnosis codes available in the patient’s medical record at discharge from the short-term acute care hospital and admission to long-term acute care.

^l^N/A: not applicable; data was not readily available through retrospective review.

### Outcomes

Using LOS as a read out for disease severity (ie, the more severe the COVID-19 illness, the longer the LOS in rehabilitation), regression analyses were performed to determine if patient sex, age, or BMI affected LOS, all of which have been noted to increase the risk of severe or prolonged COVID-19 illness [23,24]. Examining age as a potential risk factor for longer LOS and prolonged COVID-19 rehabilitation, we observed a positive correlation among COVID-19 patients (r^2^=0.05; *P*=.02; Figure 3B). No such correlation was observed in the reference cohort (data not shown). When each sex was analyzed separately, we observed a positive correlation between LOS and age among males in the COVID-19 cohort (r^2^=0.07; *P*=.02; Figure 3C), but not among females (r^2^=0.001; *P*=.55; Figure 3D).

**Figure 4:**
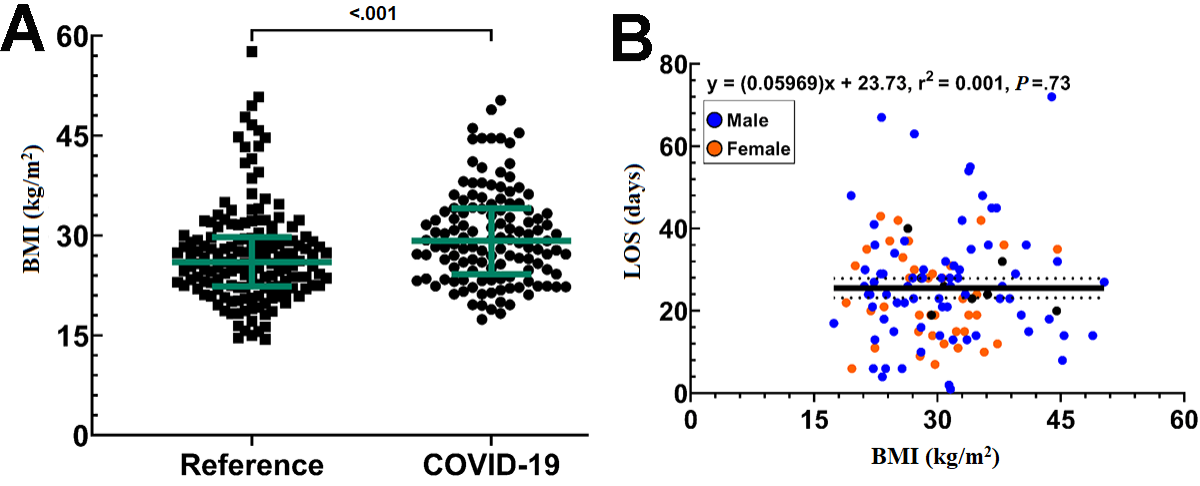
BMI as a risk factor for prolonged COVID-19 illness. (A) Scatter plot showing the distribution of BMI in the reference and COVID-19 cohorts. Lighter colored lines represent the median and interquartile range. (B) Nonlinear regression analysis showing the correlation between COVID-19 patient BMI and long-term acute care hospital length of stay (LOS). Solid regression lines show the correlation coefficient surrounded by the 95% CI as dotted lines.

BMI was greater in the COVID-19 cohort than in the reference cohort (Figure 4A, Table 1). Using regression analysis, no correlation was observed between BMI and LOS overall in the COVID-19 cohort (r^2^=0.001; *P*=.73; Figure 4B). Similarly, no such correlation was observed for males or females separately in the COVID-19 cohort (data not shown) or in the reference cohort (data not shown).

#### Respiratory Therapy

**Figure 5:**
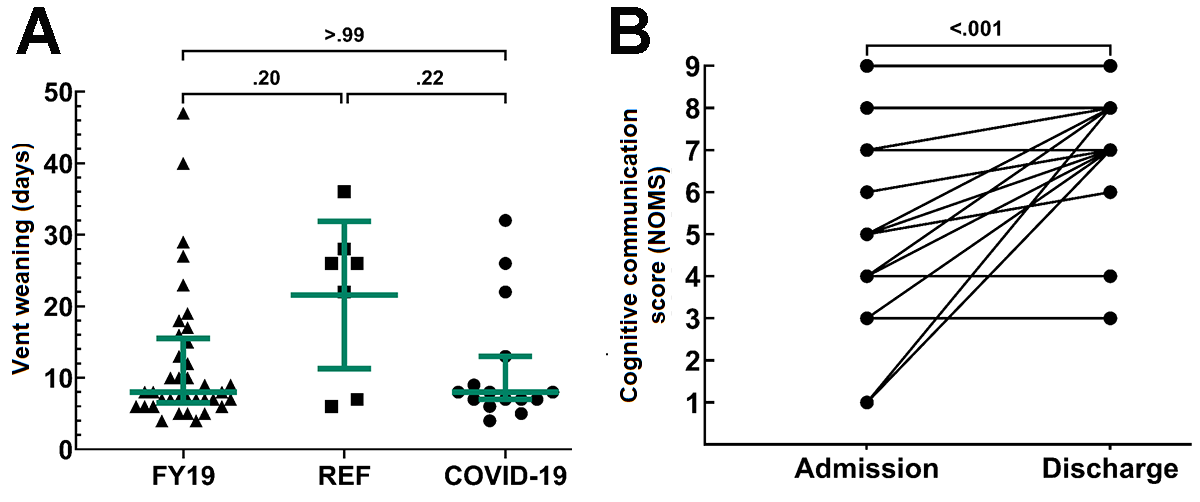
COVID-19 patient respiratory and cognitive-communication outcomes. (A) Scatter plot showing the comparison of ventilator wean times among patients mechanically ventilated during fiscal year 2019 (October 2018 through September 2019) (n=37), the reference cohort (n=7), and the COVID-19 cohort (n=15). The colored lines represent the median and interquartile range. (B) Evaluation of the cognitive communication score of COVID-19 patients recommended for speech-language pathology services (n=75) at admission and discharge. NOMS: National Outcomes Measurement System.

Of the 43 patients admitted with a tracheostomy, 37.2% (16/43) required mechanical ventilation and 62.8% (27/43) did not; 93.8% (15/16) of mechanically ventilated patients in the COVID-19 cohort were weaned by the data cutoff. Compared to the reference cohort, the mean ventilator wean time in the COVID-19 cohort tended to be shorter (21.5, 95% CI 11.3-31.9 vs 11.3, 95% CI 6.6-15.9 days; *P*=.23). Given the small number of patients being mechanically ventilated in the reference cohort (n=7), we also compared the COVID-19 cohort wean time to that of all patients for fiscal year 2019 (ie, October 2018 through September 2019) in the LTACH (12.2, 95% CI 8.9-15.5 days; n=37) and found no difference between the 2 groups (*P*>.99; Figure 5A). For those weaned from mechanical ventilation, it was an additional mean duration of 15.1 (SD 13.3) days until tracheostomy decannulation. In comparison, for those not mechanically ventilated, the mean time from admission to tracheostomy decannulation was 16.3 (SD 11.4) days.

#### Speech-Language Pathology

In the COVID-19 cohort, 59% (75/127) of admissions were recommended for speech-language pathology evaluation. Of those, 81% (61/75) were admitted with a modified diet or instructions for nothing by mouth or nil per os (NPO). Following a dysphagia evaluation, most patients were upgraded from NPO to a regular consistency diet. At discharge, 75% (56/75) of patients were consuming a regular consistency diet. Further, 49% (37/75) of patients evaluated by a speech-language pathologist were admitted with a tracheostomy, with or without mechanical ventilation, and 73% (27/37) were found to have some form of voicing disorder, including aphonia (13/37), dysphonia (13/37), or dysarthria (1/37). At discharge, only 35% (13/37) of patients had voicing limitations.

Speech-language pathologists also evaluated patients for cognitive-communication deficits using the modified NOMS scale shown in Table 2. At admission, 58% (44/75) of patients were rated as either baseline or within functional limits, 37% (28/75) were found to have impairments ranging from mild to severe, and 4% (3/75) could not be assessed. The mean cognitive-communication score at admission was 7.2 (95% CI 6.7-7.6). Deficits primarily affected the areas of attention, processing speed, short-term memory, and complex executive functioning skills. Many patients showed improvement by discharge, with 72% (54/75) being at baseline or within functional limits; 21.3% (16/75) having only mild residual cognitive deficits needing minimal cues or memory aides for maintaining attention, completing tasks, or problem solving; and 6.7% (5/75) continuing with moderate-to-severe cognitive deficits. At discharge, the mean cognitive-communication score was 7.8 (95% CI 7.6-8.0), which is a modest yet significant improvement from admission (*P*<.001; Figure 5B). Continued speech-language pathology services were recommended for 39% (29/75) of patients after discharge. Due to the retrospective nature of the study, similar values were not readily available for the reference cohort and were not included.

**Table 2:** Cognitive-communication status scoring in the COVID-19 cohort.

Description		Admission (N=75)		Discharge (N=75)
**Scoring^a^, n**				
	Unable to assess (score 1)	3	0	
	Profound impairment (score 2)	0	0	
	Severe impairment (score 3)	2	1	
	Moderate-severe impairment (score 4)	3	1	
	Moderate impairment (score 5)	5	0	
	Mild-moderate impairment (score 6)	3	3	
	Mild impairment (score 7)	15	16	
	Within functional limits (score 8)	28	38	
	Baseline (score 9)	16	16	
Mean score (95% CI)		7.2 (6.7-7.6)^b^		7.8 (7.6-8.0)^b^

^a^To better analyze patient outcomes, a modified National Outcomes Measure System scale was used for speech-language pathology cognitive-communication status evaluations.

^b^The nonparametric Wilcoxon matched pairs test was used, and the group difference (based on differences of the means) was 0.64 (95% CI 0.30-0.98; *P*<.001).

#### Physical and Occupational Therapy

Due to wheelchair dependence prior to STACH admission, emergent readmission to a STACH, continuing care at the time of data cutoff, and incomplete data collection, complete (ie, admission and discharge) gait and functional status data (FIM scores and gait distance) were only available for 99 of 127 total COVID-19 admissions and 90 of 170 reference cohort admissions. At admission, 44% (40/90) of patients in the reference cohort and 53% (52/99) of patients in the COVID-19 cohort were unable to ambulate or required maximum assistance (Table 3). The majority of patients in both the reference (69/90, 77%) and COVID-19 (88/99, 89%) cohorts displayed functional status improvement from admission to discharge, with many patients showing an increase in functional ability by 4 or more levels. These measurements were then evaluated using 2×2 two-way mixed effects ANOVA tests.

**Table 3:** Functional Independence Measure assistance scoring for ambulation.

Description		Reference cohort		COVID-19 cohort	
		Admission (N=90)	Discharge (N=90)	Admission (N=99)	Discharge (N=99)
**Scoring^a^, n (%)**					
	Unable/dependent^b^ (score 1)	36 (40)	19 (21)	51 (52)	11 (11)
	Maximal assistance^c^ (score 2)	4 (4)	2 (2)	1 (1)	0 (0)
	Moderate assistance^d^ (score 3)	6 (7)	3 (3)	6 (6)	3 (3)
	Minimal assistance^e^ (score 4)	36 (40)	18 (20)	35 (35)	14 (14)
	Supervision^f^ (score 5)	8 (9)	30 (33)	6 (6)	29 (29)
	Modified independence^g^ (score 6)	0 (0)	13 (14)	0 (0)	28 (28)
	Independence^h^ (score 7)	0 (0)	5 (6)	0 (0)	14 (14)
Mean score (95% CI)		2.7 (2.4-3.1)^i^	4.1 (3.7-4.7)^i^	2.4 (2.1-2.7)^i^	4.9 (4.6-5.3)^i,j^

^a^To track patient functional ability, Functional Independence Measure scoring was used to assess the level of assistance required for ambulation at patient admission and discharge.

^b^Patient is either unable to ambulate or is only able to perform 24% of activity.

^c^Patient can perform 25%-49% of activity.

^d^Patient can perform 50%-74% of activity.

^e^Patient can perform at least 75% of activity.

^f^Patient does not need physical assistance but does require hands-on guidance, supervision for safety, cueing, coaxing, or set up.

^g^Patient does not need the physical presence of a second person, but requires equipment or takes more than reasonable time, or there are safety concerns.

^h^Patient does not require any equipment or the physical presence of a second person.

^i^The Šídák multiple comparisons test was used to compare in-group differences (based on differences of the means) between admission and discharge. The group difference was 1.3 (95% CI −1.7 to −1.0; *P*<.001) in the reference cohort and 2.5 (95% CI −2.8 to −2.2; *P*<.001) in the COVID-19 cohort.

^j^Significantly different compared to the mean discharge Functional Independence Measure score in the reference cohort; mean difference is −0.841 (95% CI −1.39 to −0.297; *P*=.001)*.*

For ambulation FIM scores, the mean ambulation assistance scores increased in both the reference (2.73, 95% CI 2.4-3.1 to 4.1, 95% CI 3.7-4.7) and COVID-19 (2.4, 95% CI 2.1-2.7 to 4.9, 95% CI 4.6-5.3) cohorts (Table 3). Two-way mixed effects ANOVA showed a significant main effect associated with time (*F*_1,187_=335.7; *P*<.001) on FIM scores, with overall discharge scores (mean=4.498) being greater than admission scores (mean=2.584). Although we also observed a significant interaction effect between time and cohort designation (*F*_1,187_=29.78; *P*<.001), we did not observe a significant main effect of cohort designation (*F*_1,187_=1.538; *P*=.22) on FIM scores alone. The pooled mean FIM score of the reference cohort (mean=3.406) was marginally lesser than that of the COVID-19 cohort (mean=3.677).

**Figure 6:**
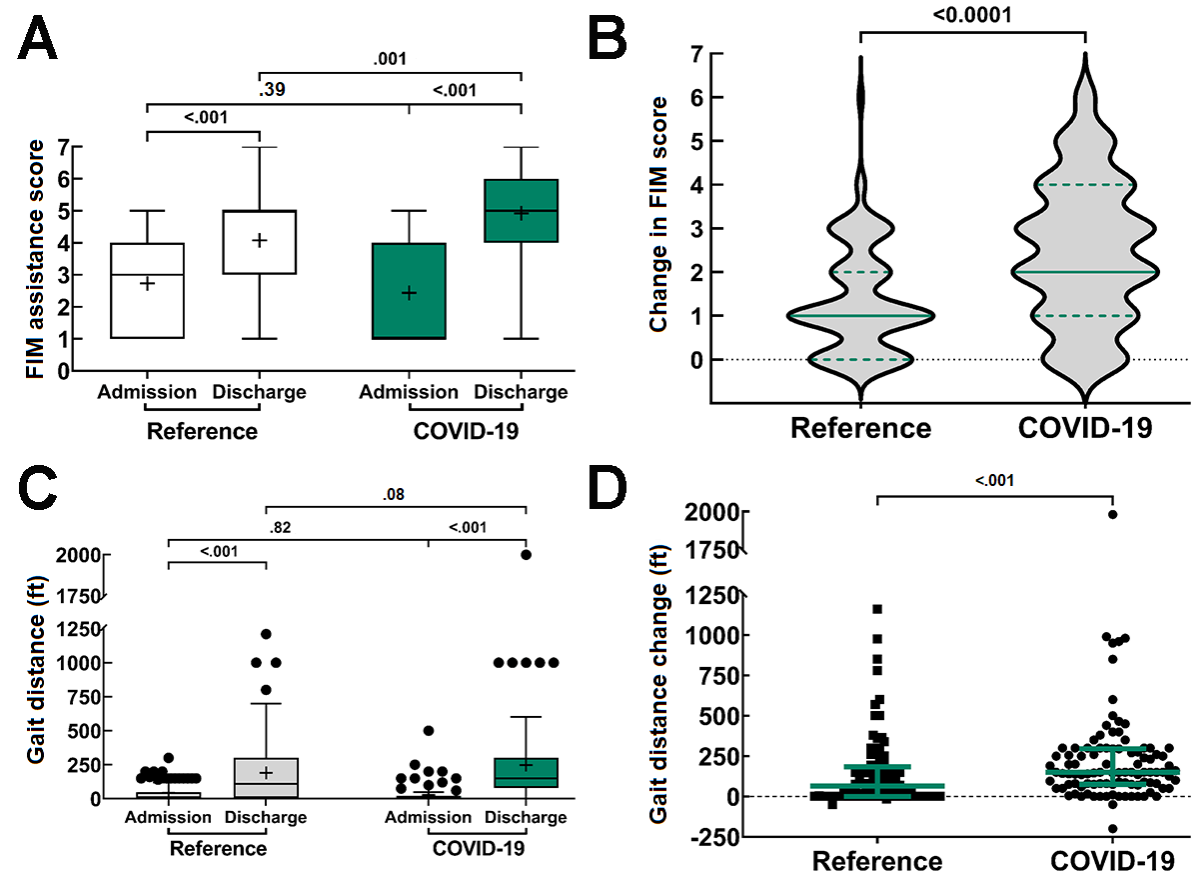
Functional Independence Measure (FIM) assistance scores and gait distances as measures of functional ability. (A and C) For both the reference (n=90) and COVID-19 (n=99) cohorts, FIM assistance scores and gait distances were collected at admission and discharge. In-group and between-group comparisons were made using the Šídák multiple comparisons test following a 2×2 two-way mixed effects analysis of variance test for main effects associated with group and time. Box plots represent the median and the 25% and 75% quartiles. The whiskers extend 1.5 and -1.5 of the interquartile range; circle symbols reflect data points beyond the 1.5 interquartile ranges; and the “+” symbol represents the mean. (B and D) Changes in FIM assistance scores and gait distances were then compared using a nonparametric Mann-Whitney U test. B, Violin plot with medium smoothing to show the distribution of FIM score changes; the colored lines represent the median and interquartile range. D, Scatter plot, with the colored lines representing the median and interquartile range.

Using the Šídák multiple comparisons test, we then tested to see what in-group and between-group comparisons were significantly different. In-group comparisons for both cohorts showed a significant increase in FIM scores between admission and discharge (*P*<.001), further highlighting the main time effect noted in the 2-way mixed effects ANOVA (Figure 6A; Table 3). Between-group comparisons revealed that, with a mean difference of 0.299 (95% CI −0.245 to 0.843), there was no difference in FIM scores at admission between the 2 cohorts (*P*=.39) (Figure 6A; Table 3). This indicates that patients in both cohorts required the same or similar levels of assistance at admission. Comparing the FIM scores at discharge revealed that, with a mean difference of −0.841 (95% CI −1.39 to −0.297), the mean discharge FIM scores were significantly greater in the COVID-19 cohort than in the reference cohort (*P*=.001) (Figure 6A; Table 3). Together, we interpret these data to indicate that while both cohorts had similar FIM scores at admission and both improved over time, the discharge FIM scores were greater in the COVID-19 cohort than in the reference cohort.

We then compared the mean FIM assistance score change from admission to discharge in the 2 groups. The score change was greater in the COVID-19 cohort than in the reference cohort (2.5, 95% CI 2.2-2.8 vs 1.3, 95% CI 1.1-1.6 points; difference of medians=1.0, 95% CI 1.0-2.0; *P*<.001; Figure 6B).

The same analysis was conducted for gait distance (feet). The mean gait distance increased in both the reference (43.3, 95% CI 29.8-57.0 to 189.9, 95% CI 139.0-240.8 feet) and COVID-19 (27.5, 95% CI 14.1-40.9 to 248.7, 95% CI 191.1-306.4 feet) cohorts (Table 4). Two-way mixed effects ANOVA showed a significant main effect associated with time (*F*_1,187_=97.15; *P*<.001) on gait distance, with the pooled discharge distance (mean=219.3 feet) being greater than the pooled admission distance (mean=35.5 feet). Although a significant interaction effect was observed between time and cohort designation (*F*_1,187_=4.02; *P*=.046), a significant main effect related to cohort designation (*F*_1,187_=0.9994; *P*=.32) on gait distance was not observed, with the pooled gait distance being marginally lesser in the reference cohort (mean=116.7 feet) than in the COVID-19 cohort (mean=138.1 feet).

**Table 4:** Gait distance at patient admission and discharge.

Variable	Reference cohort	COVID-19 cohort	Between-group difference, mean (95% CI); *P* value
Admission distance (feet)^a^, mean (95% CI)	43.4 (29.8 to 57.1)	27.53 (14.1 to 40.9)	15.9 (−48.0 to 79.8); *P*=.82^b^
Discharge distance (feet)^a^, mean (95% CI)	189.9 (139.0 to 240.8)	248.7 (191.1 to 306.4)	−58.9 (−122.7 to 5.0); *P*=.08^b^
Within-group difference (feet), mean (95% CI); *P* value	−146.4 (−207.3 to −85.6); *P*<.001^b^	−221.1 (−279.2 to −163.2); *P*<.001^b^	74.8 (2.0 to 147.6); *P*<.001^c^

^a^Complete admission and discharge gait distances were only available for a subset of the total admissions for both the reference (n=90) and COVID-19 (n=99) cohorts.

^b^Calculated using the Šídák multiple comparisons test following a mixed effects analysis of variance.

^c^Comparison of group differences calculated using the Mann-Whitney *U* test.

Using the Šídák multiple comparisons test, we again tested to see what in-group and between-group comparisons were significantly different. In-group comparisons for both cohorts showed a significant increase in gait distance between admission and discharge (*P*<.001), further highlighting the main time effect noted in the 2-way mixed effects ANOVA (Figure 6C; Table 4). Between-group comparisons showed that, with a mean difference of 15.9 (95% CI −48.0 to 79.8), there was no difference in gait distance at admission between the 2 cohorts (*P*=.82) (Figure 6C; Table 4). This indicates that patients in both cohorts were able to ambulate the same or similar distances at admission. Comparing the gait distances at discharge revealed that, with a mean difference of −58.9 (95% CI −122.7 to 5.0), the mean discharge gait distances were nearly significantly greater in the COVID-19 cohort than in the reference cohort (*P*=.08).

Further, we compared the mean change in gait distance from admission to discharge in the 2 groups. The gait change was greater in the COVID-19 cohort than in the reference cohort (221.2, 95% CI 164.8-277.6 vs 142.5, 95% CI 95.9-189.1 feet; difference of medians=90, 95% CI 25.0-100.0; *P*<.001; Figure 6D; Table 4).

#### Additional Wound Care, Physical Therapy, and Medical Service Considerations

With prone positioning becoming the standard of care for COVID-19–related respiratory failure and pneumonia, many patients in the COVID-19 cohort developed atypical facial pressure injuries during their STACH stay. Patients in the COVID-19 cohort were admitted with approximately 69 total body pressure injuries (stage 3 or 4) requiring consultation; 30% were located on the face, usually on both cheeks, with one more severe than the other and having thick eschar development. Conservative treatment without sharp debridement resolved most cases of facial pressure injuries. New injuries were prevented by implementing adhesive foam cushioning to facial pressure areas. Patients were also likely more hemodynamically stable during LTACH care and therefore somewhat less likely to develop pressure injuries.

Unilateral and bilateral wrist and foot drop were also observed in some patients, potentially due to prolonged prone positioning in the STACH causing peripheral nerve compression. Patients with wrist drop showed some improvement, though some required orthoses or occupational therapy after discharge. Some COVID-19 patients presented conditions atypical for respiratory diseases, such as neurological findings, peripheral nerve injuries, paresthesia, and cognitive impairment. Neurological symptoms may have resulted from the use of paralytics or prolonged prone positioning during STACH treatment [12,25].

## Discussion

### Principal Findings

The emergence of the novel coronavirus SARS-CoV-2 has resulted in a worldwide pandemic with 281 million infections and 5.4 million deaths as of this writing [26]. For other facilities to reference now or in the future when treating patients with COVID-19, the goal of this retrospective study was to summarize and report the observations, experiences, and methods used by clinicians at our LTACH and how these practices impacted patient outcomes. Using our holistic treatment strategy, we focused on all aspects of patient recovery, with the majority of our patients with severe active COVID-19 or post–COVID-19 showing significant improvement through this coordinated care.

During the study period, 93% of patients admitted on mechanical ventilation were weaned, and 96% of patients admitted with a tracheostomy without mechanical ventilation were decannulated. Though many patients had functional limitations and were nonambulatory at admission, the COVID-19 cohort showed significant functional improvement by discharge, including a 149% greater change in gait distance travelled compared to the reference cohort. While both cohorts had similar FIM assistance scores at admission and both improved over time, the FIM assistance scores of the COVID-19 cohort were significantly greater than those of the reference cohort at discharge. Patients receiving speech-language therapy also showed improvements during their LTACH treatment, with 40.5% fewer patients having voicing limitations at discharge and only 28% having residual cognitive-communication deficits. Together, these observations indicate the potential benefits of individualized, focused, and holistic rehabilitation in a population severely affected by COVID-19 [27].

Though not significant, the COVID-19 cohort ventilator wean time (10.4 days) was shorter than historical facility wean times (12.2 days in 2019, 20.6 days in 2018, and 14 days in 2017) [28]. Based on our clinical observations, the COVID-19 cohort generally presented fewer complicated pulmonary and cardiac comorbidities than typical patients with tracheostomy, with or without mechanical ventilation. This may have contributed to the shorter ventilator wean time. These observations support the idea that pulmonary rehabilitation could play an important role in COVID-19 treatment and recovery [29]. Further, compared to patients with chronic pulmonary conditions, the COVID-19 cohort patients, who were generally new to respiratory deficits, improved rapidly with appropriate respiratory management.

In regard to patient susceptibility and risk for severe COVID-19 illness, we observed a positive correlation between patient age and patient LOS. In contrast to what has been reported, we did not observe a correlation between patient BMI and disease severity/LOS [23,24]. These differences could be attributed to several factors, including better pre–COVID-19 health status compared to that of patients typically cared for at the facility, current employment status at the time of COVID-19 diagnosis (many of the patients in the cohort were health care workers or first responders), and motivation to return home (as visitation was restricted).

The quick progression in cognitive-communication skills during LTACH stay was also likely multifactorial, involving discontinuation of sedatives, improved metabolic status, awareness of deficits, and an ability for patients to carry over compensatory strategies learned in therapy. However, ongoing cognitive-communication impairment is possible in patients who have had COVID-19, and these individuals may benefit from continued therapy services after discharge [30].

Many of our patients were admitted on a modified diet or NPO because of their inability to participate in swallowing assessments at acute care, the severity of their medical condition, or limited access to instrumental assessments during speech-language pathology evaluations due to droplet precautions. The prompt advancement of diet in the LTACH setting was mostly the result of clinical swallowing evaluations showing minimal residual weakness within the oropharyngeal swallowing mechanism. Therefore, it is possible to largely rely on clinical swallowing evaluations for patients with COVID-19, thus minimizing the risk of viral exposure by limiting aerosol-generating instrumental assessments [27]**.** To protect from aerosols when assessing patients with unknown or suspected positive SARS-CoV-2 status, speech-language pathologists should consider the continued use of clear face masks, face shields, and other eye protection during therapy sessions.

Patients also likely received emotional benefit from the formation of inpatient COVID-19 support groups. These groups, facilitated by a physical therapist and a social worker, were a collaborative effort to provide patients who were recovering from COVID-19 with the opportunity to speak with other patients experiencing similar concerns during their hospital course. With guidance from the group facilitators, patients were encouraged to ask questions and share their experiences in an open discussion format, which ultimately generated insightful feedback for the staff on patient care during the pandemic. Conversation topics focused on processing the initial illness onset and acute hospital stay; acknowledging and learning to cope with their physical, respiratory, emotional, and social changes; and preparing for their future after LTACH discharge. Participation was capped at 6 patients per meeting, and multiple meetings were convened as necessary to accommodate all interested patients.

### Limitations

When evaluating these findings, several limitations need to be considered. First, as this was a retrospective study, *a priori* power analysis and sample size estimation were not conducted. Further, as this was a single-center study, the findings may or may not fully reflect the expected findings of other LTACHs or similarly structured institutions. Additionally, in an effort to create a reference for comparison of this unique population, a retrospective historical control was used. As such, all outcome measures could not be compared (ie, the NOMS was only readily available for the COVID-19 population). This also resulted in the population sizes being uneven despite the COVID-19 data being collected over 5 months versus 3 months for the reference.

There is also a possibility that the COVID-19 cohort received treatment at a slightly less intensity due to initial droplet precautions and isolation to the room. However, due to similarities in the baseline status (ie, assistance scores and gait distances), we are confident the populations were generally comparable as department standards for treatment and therapy doses for medically complex patients were followed in both cohorts.

It needs to be considered that the best treatment practices were actively being developed and implemented during the study period. Thus, the first COVID-19 patients admitted and treated may not have benefited from the knowledge gained over time. For example, as testing guidelines, isolation procedures, and intubation and ventilation recommendations changed, so did the treatment practices.

This study is strengthened by the breadth of quantitative outcomes and the detailed descriptions of potential presentations and complications that can be expected for patients with COVID-19 being treated in a LTACH setting. The goal of this study was to discuss typical symptom presentation and recovery patterns for the COVID-19 population in the LTACH setting so as to guide treatment planning choices at other similar facilities.

### Mitigating SARS-CoV-2 Transmission in the Non–COVID-19 Patient Population

Patients cared for at LTACHs typically have complex medical conditions and are at increased risk for infection and fever; thus, there was a pressing need to isolate any potential source of SARS-CoV-2. Despite what symptoms have been described as “typical” COVID-19 symptoms, patients presented with a spectrum of respiratory symptoms, ranging from asymptomatic to respiratory distress. Consequently, all febrile patients were required to undergo SARS-CoV-2 testing and were isolated with droplet precautions until ruled out. With only 1 exception, all non–COVID-19 patients tested negative for SARS-CoV-2 during this time, indicating that our protocols effectively isolated the 37 patients who were admitted with active SARS-CoV-2 infections. Our observation supports preemptive testing in LTACHs and other health care facilities to lower the incidence of SARS-CoV-2 transmission [31]. Given the documented issues of SARS-CoV-2 transmission in some long-term care facilities, it is possible to imagine what the alternative may have been without preemptive testing [32-34].

One limiting aspect of care during this period of the pandemic was the length of time it took to obtain SARS-CoV-2 test results for patients who were admitted with an active infection, so they could come off droplet precautions, which was over 2 months in many cases [35]. On May 20, 2020, Connecticut Department of Public Health released a memo supporting their agreement with the findings of the Centers for Disease Control and Prevention that the live virus was undetectable after 9 days of infection, allowing for the use of a symptom-based strategy rather than a test-based strategy [36,37]. We implemented a more conservative approach, requiring at least 14 days since diagnosis and 5 days without fever or evolving symptoms. Further, given the low facility infection rate of the non–COVID-19 population, the facility policy changed around the same time from transferring patients under investigation to the COVID-19 floor, to ruling-out in place with the use of droplet precautions and a portable room air scrubber. Coming off droplet precautions was instrumental in getting patients out of their rooms and having full access to therapy.

It was also evident early on that regular, clear, and transparent communication was, and still is, vital for staff acceptance of the constantly changing situation, guidelines, and personal protective equipment protocols. To support this, department directors and managers devoted time each day to discussing COVID-19–related patient issues. These directors then met weekly with key staff members to further discuss the issues and disseminate information. Further, emails were frequently sent to all employees detailing COVID-19–related changes, statistics, and other topics of interest. In-person communication was also helpful in correcting rumors and serving as a forum for establishing best practices in the ever-changing situation.

### Conclusion

To alleviate crowded and overwhelmed STACH facilities, we envision the strategic use of LTACHs earlier in a patient’s hospital course to treat and rehabilitate those with severe COVID-19. With a greater understanding of rehabilitation progression, clinical care can be adapted to maximize the recovery of this population.

## Appendix

Multimedia Appendix 1 Gaylord Hospital tracheostomy decannulation protocol.

Multimedia Appendix 2 Gaylord Hospital mechanical ventilation weaning protocol.

## Abbreviations

ANOVA: analysis of variance
FIM: Functional Independence Measure
LOS: length of stay
LTACH: long-term acute care hospital
NOMS: National Outcomes Measurement System
NPO: nil per os (nothing by mouth)
OR: odds ratio
STACH: short-term acute care hospital
