# Supplementary material for: Patient Outcomes and Lessons Learned From Treating Patients With Severe COVID-19 at a Long-term Acute Care Hospital: Single-Center Retrospective Study
Source: JMIR Rehabil Assist Technol. 2022 Feb 10;9(1):e31502. doi: 10.2196/31502 (PMC8834875; doi:10.2196/31502)
Supplement: Multimedia Appendix 1 [file rehab_v9i1e31502_app1.docx]

**Gaylord Hospital tracheostomy decannulation protocol**

- All patients undergoing decannulation will need to be evaluated by a Pulmonary or Critical Care Medicine consultant. The consultant will document the patient is ready to go ahead with the decannulation protocol.
- After evaluation, the Pulmonary consultant may proceed with decannulation off protocol with clear documentation to the reasons why.
- For patients unable to perform cough peak flow procedure, refer to the protocol designated for inadequate peak flow.
- Daily progress of the decannulation process will be documented by the Respiratory Care Practitioner (RCP) and conveyed to covering therapists during change of shift report.
- The RCP will maintain daily communication with the LIP and/or consultant of the progress of the decannulation process.
- Once the decannulation protocol has been completed, the RCP will notify the LIP of completion of the protocol, and the LIP will enter the decannulation order in Meditech. Please refer to the section on trach removal.

I. Criteria for Initiation of Protocol:

All patients must have all of the following: Afebrile; Hemodynamically stable; Clear or stable Chest X-ray; Controlled Secretions; Peak Cough Flow ≥160 L/m; Satisfactory on-going nutrition (low risk of aspiration); No clinical evidence of tracheal obstruction; Ability to tolerate a speaking valve.

Morning ABG immediately upon completion of the NOT study will be required.

The Respiratory Care Practitioner will determine that the criteria have been met. If the patient fails to meet the criteria, the LIP notified, and the protocol cancelled.

II. Respiratory Care Practitioners/Registered Nurses:

For ALL patients:

- Decrease tracheostomy cannula size to ≤ 6mm.
  - The first tracheostomy change (post initial surgical tracheostomy insertion), change will be done by an Otolaryngology, Pulmonary, or Critical Care Medicine physician.
- Nursing to monitor and record HR, BP, and temp Monitor VS q 4 hours for 24 hours with each tracheostomy change
- RT to monitor and record O_2_ saturation, ETCO_2_, and RR q4 hours for 24 hours with each trach change
- Observe trach site for bleeding
- RCP does trach change and documents in the Clinical Information System.
- RCP/RN will notify LIP if VS become unstable and/or O_2_ Sat ≤ 92%

III. Criteria to begin plugging trials:

- VS stable (within 15% of baseline)
- For patients with no underlying lung disease: O_2_ saturation ≥ 92% and ETCO_2_ ≤ 45 mmHg
- For patients with underlying lung disease: Stable blood gases; With adequately compensated PCO_2_ and pH; O_2_ saturation ≥ 92% on room air or oxygen

IV. Decannulation

Day 1

- Plug tracheostomy
- Nursing to monitor and record HR, BP, and temp q 4 hours
- RT to monitor and record O_2_ saturation, ETCO_2_, and RR q 4 hours
- Plug up to 16 hrs, remove for sleep

Criteria for unplugging sooner than 16 hours if any of the following occur: Change in hemodynamics (BP 15% above or below baseline); Increased RR (15% above baseline); O_2_ Sat < 92%; Increase in ETCO_2_ of >20% over baseline; Increased HR (15% above baseline); Fever ≥ 100.8°F; Increase in pulmonary secretions; Stridor.

RCP documents plugging trial in the decannulation protocol documentation intervention and patient response/compliance in notes section of Clinical Information System.

Day 2

If successful with day 1 of 16 hour plugging, on day 2 plug trach and leave for 24 hours.

- Nursing to monitor and record HR, BP, and temperature q 4 hours
- RT to monitor and record O_2_ saturation, ETCO_2_, and RR q 4 hours
- Obtain overnight oximetry
- Obtain early am ABG according to criteria in section II

Day 3 (if required)

- If clinically necessary, may continue with tracheostomy plug ATC
- Nursing to monitor and record HR, BP, and temperature q 4 hours
- RT to monitor and record O_2_ saturation, ETCO_2_, and RR q 4 hours
- Obtain overnight oximetry (if not done previously)
- Obtain early am ABG according to criteria in section II (if not done previously)

Criteria for unplugging before 24 hrs: Any items listed in Section IV

RCP documents plugging trial and if appropriate that patient meets criteria for removal in notes section of the Clinical Information System.

If plugging trial fails RCP will notify LIP.

Any unsuccessful decannulation requiring replacement of the tracheostomy tube will require an Otolaryngology consult.

If a patient is moved to another unit as part of the decannulation process, RT staff will complete bedside hand-off of report.

Criteria for tracheostomy removal:

- Decannulation will take place prior to 5pm unless approved by LIP
- Stable vital signs (within 15% of baseline)
- Oxygen saturation ≥ 92%
- ETCO_2_ remains stable (within 5% of baseline)
- Overnight oximetry results **without** suggestion of sleep apnea.
- Overnight oximetry results **with** suggestion of sleep apnea:
  - RCP will leave trach in place and discuss further action with the LIP

If decannulation is decided, then the RCP will decannulate patient and place and securely tape sterile gauze over the patient’s stoma.

RCP will then document procedure and patient response in the clinical information system.

Following decannulation, all patients will be monitored with pulse oximetry for the first 24 hours post decannulation, with vital signs monitored and recorded every 4 hours for 24 hours by nursing and RT (see above). If the patient is on telemetry during the decannulation process, he/she will be maintained on telemetry.

The LIP will be notified if patient experiences:

- Change in hemodynamics (BP 20% above or below baseline)
- Increased RR (15% above baseline)
- O_2_ saturation < 92%
- Increase in ETCO_2_ of >5% above baseline
- Increased HR (15% above baseline)
- Fever ≥ 100.8°F
- Increase in pulmonary secretions
- Development of stridor

If stable, patient may be discharged 48 hours after decannulation.
