# Supplementary material for: Patient Outcomes and Lessons Learned From Treating Patients With Severe COVID-19 at a Long-term Acute Care Hospital: Single-Center Retrospective Study
Source: JMIR Rehabil Assist Technol. 2022 Feb 10;9(1):e31502. doi: 10.2196/31502 (PMC8834875; doi:10.2196/31502)
Supplement: Multimedia Appendix 2 [file rehab_v9i1e31502_app2.docx]

**Gaylord Hospital mechanical ventilation weaning protocol**

To be a candidate for Spontaneous Breathing Trial, the patient must pass the following criteria:

1. **Clinical Assessment prior to Spontaneous Breathing Trial (SBT):**
   1. Successful Spontaneous Awakening Trial (respiratory therapy [RT] to confirm with nursing [RN])
   2. No hemodynamic instability (RT to confirm with RN)
   3. Assess mental status to be within range of alert, calm, mild drowsiness to mild restlessness
   4. PaO_2_/FiO_2_ greater than 200 (Obtain values from most current ABG- done within last 48 hours)
   5. PEEP ≤ 7.5
   6. FIO_2_ ≤ 0.50
   7. Patient is making efforts to breathe
   8. Secretions scant non-purulent, suctioning not more than every 4 hr
   9. Temp ≤ 100.5°F
   10. Document tracheostomy size and type

**Prior to initiation of SBT, notify cardiac monitor technician**

1. **Interventions:**
2. If patient had completed 2 hrs or more of trach mask trial at acute care, can proceed with supervised 30-minute tracheostomy mask trial. If patient remains stable, proceed with tracheostomy mask progress.
3. If patient has not performed tracheostomy mask trial, place on CPAP of +5 and PSV of +5 with a maximum FIO_2_ of 0.50 and perform RSBI (Rapid Shallow Breathing Index)
   - Respiratory rate/tidal volume (f/Vt) should be <105 to proceed with tracheostomy mask trial
4. If patient is on CPAP and remains stable, continue on current settings: PS 5, CPAP 5, FIO_2_ < 0.50. Then if these settings are tolerated for 30 minutes to 2 hours (based on overall status of patient), initiate reassessment for spontaneous breathing success. If patient continues to remain stable, begin with tracheostomy mask trials.
5. Resume previous ventilator support settings & terminate spontaneous breathing trial and inform RN/MD, if any of the following occur: Respiratory Rate > 35; Respiratory Rate <8; Oxygen saturation <88%; Respiratory distress; Mental status change; Acute cardiac arrhythmia/Hemodynamic changes; Temperature ≥ 100.5°F
6. Obtain ABG per ABG Protocol.
7. **Reassessment:**

Spontaneous breathing trial deemed successful based upon the following criteria: Stable gas exchange; Hemodynamically stable; Temperature ≤ 100.5 °F; RR < 35; SpO2 > 88% on not more than 50% FiO_2_; Positive cuff leak; Absence of diaphoresis and absence of use of accessory muscles; Ability to maintain airway with ability to clear secretions

**If patient fails any criteria in reassessment, pulmonary physician will be called, and trial will be attempted next day.**

- Assess for presence of cuff leak
- Begin 2 hr TM trial BID with intervening 2 hr rest
- Record O_2_ SAT, ETCO_2_ and end each trial
- Proceed with subsequent daily progressive increases of time on TM as follows
- Notify the pulmonary physician for proceeding with ventilator liberation if all criteria in the reassessment are met for spontaneous breathing trial.

**Tracheostomy Mask Progress:**

- 4 hrs BID (2 – 4 hrs rest interval in between)
- 8 hrs consecutive
- Up to 16 hrs consecutive, rest on ventilator support at night x 2 nights

When the patient can tolerate 16 hours x2 consecutive days with rest on ventilator support at night, proceed to 24 hours and obtain ABG after the 24-hour period. After obtaining the ABG, consult with pulmonary/critical care.
